# Foveal avascular zone area measurements using OCT angiography in patients with type 2 diabetes mellitus associated with essential hypertension

**Published:** 2019

**Authors:** Daniela Stana, Vasile Potop, Sînziana Luminiţa Istrate, Cecilia Eniceicu, Ana Raluca Mihalcea, Irena Gabriela Paşca, Abdallah Aqel, Radu Ciuluvică, Dana Moraru

**Affiliations:** * Ophthalmology Department, University Emergency Hospital, Bucharest, Romania; ** Ophthalmology Department, “Carol Davila” University of Medicine and Pharmacy, Bucharest, Romania; *** Anatomy Department, “Carol Davila” University of Medicine and Pharmacy, Bucharest, Romania; **** Academic Center for Optical Engineering and Photonics, Politehnica University, Bucharest, Romania

**Keywords:** diabetes, hypertension, foveal avascular zone

## Abstract

**Objective.** This study followed the variability in the foveal avascular zone (FAZ) area measured using optical coherence tomography angiography (OCTA) in patients with type 2 diabetes mellitus and high blood pressure.

**Material and Methods.** This prospective, non-randomized, cohort study evaluated 46 eyes in 26 patients with high blood pressure associated with diabetic non-proliferative retinopathy (mild, medium, and severe forms) and diabetic proliferative retinopathy.

**Results.** Our results showed early macular alterations (microaneurysms, leakage, neovascularizations, intraretinal microvascular abnormalities), a higher class of severity despite a relatively normal clinical aspect and higher values of FAZ associated with neovascularization undetected by SD-OCT (spectral domain-OCT).

**Conclusion.** Measurement of the foveal avascular zone area using OCTA early detects macular alterations that precede classical retinography and SD-OCT determinations.

**Abbreviations:** FAZ = foveal avascular zone; OCTA = optical coherence tomography angiography; IRMA = intraretinal microvascular abnormalities; SD-OCT = spectral domain-optical coherence tomography; OU = both eyes; PD = papillary diameter; ETDRS = early treatment diabetic retinopathy study; BP = blood pressure; OD = right eye

## Introduction

OCTA (optical coherence tomography angiography) made it possible to enlarge our knowledge of diabetic retinopathy, to quantify retinal edema and to highlight the retinal and choroidal circulation without injecting any contrast medium [**1**].

Diabetic macular ischemia and microvascular changes are complications of diabetes mellitus and studies have shown that hyperglycemia and hypertension play an important role in disease development and progression [**2**]. 

Di G et al. investigated the morphology of the foveal avascular zone (FAZ) area and the clinical study showed a larger FAZ in patients with diabetes mellitus and a much larger FAZ in patients with more severely damaged retina [**3**]. 

OCT angiography is a new non-invasive method for studying the FAZ and can provide considerable amount of data in understanding the relationship between hyperglycemia, hypertension, and changes of foveal avascular zone area in patients with diabetes mellitus. Bates NM et al. also tried to demonstrate that an earlier detection of diabetic retinopathy could be possible by using OCT-angiography and FAZ changes, allowing better visual outcomes [**4**].

In the evolution of diabetes, examination of the retina is a mandatory feature, but the early stages of diabetes do not fully benefit from classical imaging, so in the last publications of the last year it has been reported that microvascular changes in diabetes can be early detected with OCT angiography (OCTA). However, literature data requires additional angiographic studies of the risk of progression of diabetic retinopathy based on the presence of comorbidities and hypertension.

The aim of the study was to find correlations of changes in the foveal avascular zone area (FAZ) by OCT angiography (OCTA) in patients with type 2 diabetes mellitus and essential hypertension.

## Material and Methods

**a) Design of the study**

This paper presented a prospective, non-randomized, cohort, comparative study that analyzed the patients from the University Emergency Hospital, Clinic of Ophthalmology, presenting with OU: diabetic retinopathy and hypertension diagnosed and under medical treatment, between January and June 2017. The study complied with the Helsinki Convention on Patients’ Rights. The informed consent was presented and signed by all participants after presenting the study objectives.

**b) Selection of patients**

Patients were enrolled in the study according to the inclusion criteria described in **Table 1**.

The exclusion criteria consisted of retinal disorders that might interfere with the correct assessment of the progression of diabetic retinopathy or ophthalmologic disorders that might prevent good eye visualization (**Table 1**).

**Table 1:** Criteria for inclusion and exclusion of patients enrolled in the study

Inclusion criteria	Exclusion criteria
Age > 18 years old	Age < 18 years old
History of type II compensated diabetes for at least 5 years	Other associated retinal disorders
History of arterial hypertension for at least 5 years	Other associated vascular diseases (vasculitis)
Absence of media opacity	Media opacity (cataract, haemophthalmus, corneal diseases)

**c) Batch description**

We studied 26 patients, a total of 42 eyes. Patients were followed by general clinical examination and ophthalmologic examination.

**d) Ophthalmologic evaluation**

All patients were evaluated with a full ophthalmologic examination; data analyzed and quantified using the following parameters:

- Visual acuity;

- Biomicroscopic examination of anterior and posterior pole;

- Retinography photographic exam

- Macular examination by OCT angiography

Visual acuity was verified with Snellen chart and retinoscope. Biomicroscopic examination of the anterior pole was performed for each patient. Indirect examination of the posterior pole was performed using a biomicroscope and a Volk Super Field lens. The cases where a correct eye evaluation could not be performed due to the presence of crystalline or corneal opacities, retinal bleeding greater than ½ PD or haemophthalmus were excluded.

The retinophotography examination of the eye was performed in all cases with the Zeiss Fundus Camera after the pupil’s dilation. At each examination, the subtype of diabetic retinopathy and the presence of significant clinical macular edema were specified according to the ETDRS classification.

OCT angiography was performed with Avanti RTVue XR System (Optovue, Inc., Fremont, CA, USA). The OCT has an A scanning rate of 70,000 scans per second using a scanning wave centered at 840 nm with a bandwidth of 45nm [**5**].

**Fig. 1:**
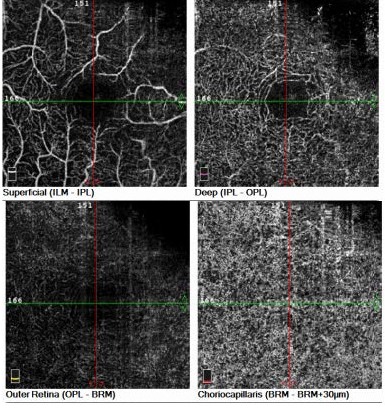
OCTA Avanti RTVue imaging of vascular layers in a newly discovered type 2 diabetic patient with no diabetic retinopathy

Each OCT angiogram contains 304 × 304 Mode A scan with two consecutive B-mode scanning, with an average acquisition time per macular volume of about 3 seconds. Two orthogonal shots were performed to reduce movement artifacts due to the patient’s breathing or blinking movements. All measurements included the measurement of the superficial and profound vascular layer, their spatial delimitation being as it follows:

• For the superficial vascular layer, a reference distance of 3 μm under the inner limiting membrane (ILM) as the upper edge and the lower margin at 15 μm under the inner plexiform layer (IPL) was taken.

• For the deep vascular layer, the boundaries were delimited at 15 μm (upper margin) and 70 μm (lower margin) under the internal plexiform layer (**Fig. 1**).

At each examination, both systolic and diastolic blood pressure was evaluated by two consecutive measures at a distance of 30 minutes apart.

**e) Data processing**

In the study, the statistical analysis and graphical representations were made using the SPSS 20 (Statistical Package for Social Sciences) program. Analysis of the statistical data was done on a sample of 26 people - 42 eyes [**6**-**8**].

## Results

Patients were divided according to ETDRS (early treatment diabetic retinopathy study) criteria into group A with mild diabetic non-proliferative retinopathy (16 eyes) and medium diabetic non-proliferative retinopathy (10 eyes) and group B with severe non-proliferative diabetic retinopathy (15 eyes) or proliferative diabetic retinopathy (11 eyes). At baseline, BP presentation in group A was 150/ 75 mm Hg and in group B 160/ 85 mm Hg. Quantitative analysis of FAZ in group B was 0.479 mm2 +/ - 0.053 mm2 and FAZ in group A was 0.257 mm2 +/ - 0.043 mm2. FAZ quality analysis revealed numerous undetected subclinical changes in SD-OCT or in standard retinophotography (microanalysis and leakage, neovascularization, IRMA) that led to the inclusion of patients with an aggressive aspect of diabetic retinopathy in OCTA in a “high risk” class of retinopathy progression. 

**Fig. 2:**
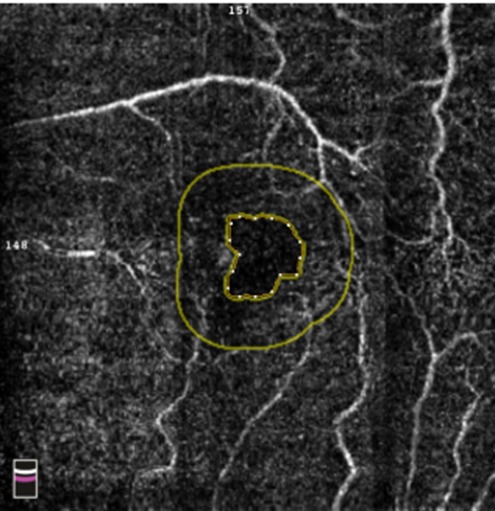
OCT Avanti RT Vue image (Optovue, USA) of the foveal avascular zone area in a patient with mild non-proliferative diabetic retinopathy which did not show enlargement of the foveal avascular zone (FAZ 0.171 mm2)

**Fig. 3:**
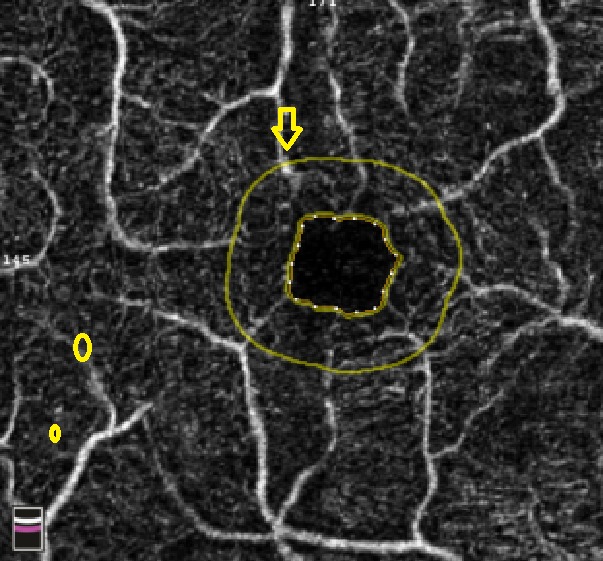
OCTA Avanti RTVue image of the foveal avascular zone area of a patient with uncontrolled type 2 Diabetes, OD (right eye) medium and severe non-proliferative diabetic retinopathy and hypertension. There is a widening of the foveal avascular zone (FAZ 0.257 mm2), microaneurysms (yellow circle) and IRMA-intraretinal microvascular abnormalities (yellow oval) as well as venous dilation

**Fig. 4:**
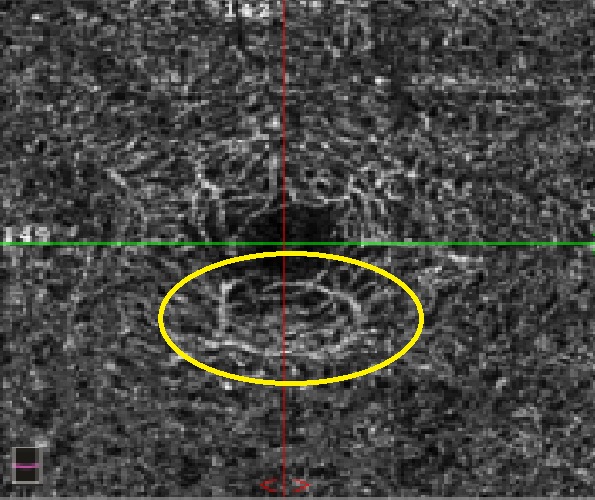
OCTA Avanti RTVue image of the patient’s deep vascular layer according to ETDRS criteria presented with non-proliferative diabetic retinopathy. However, the presence of inferior parafoveolar neovascularization, which implies grading of proliferative diabetic retinopathy, was observed

**Fig. 5:**
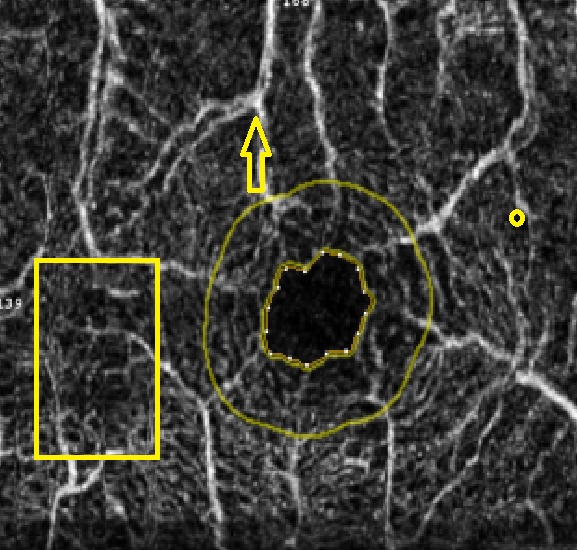
OCTA Avanti RTVue image in a patient with medium non-proliferative diabetic retinopathy, which shows a non-perfusion area (yellow patch), venous dilation (yellow arrow), and presence of IRMA (yellow circle). The foveal avascular zone has enlarged dimensions (0.220 mm2)

**Fig. 6:**
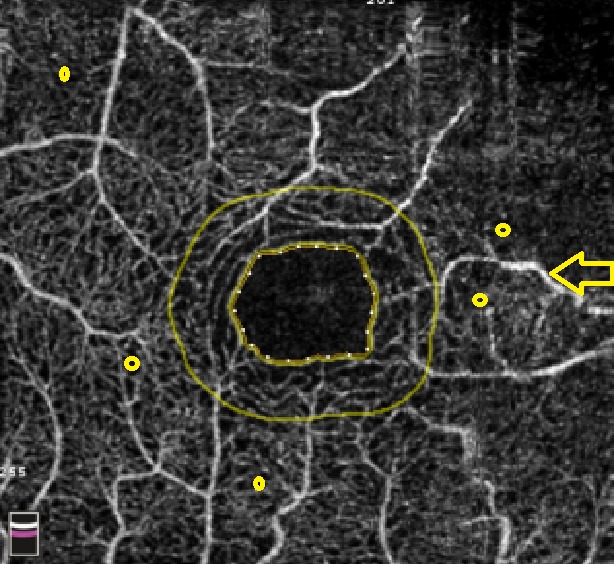
OCTA Avanti RTVue image of the foveal avascular zone area of a patient with uncontrolled type 2 diabetes, severe non-proliferative diabetic retinopathy, and severe hypertension. There is a widening of the foveal avascular zone compared to normal value (FAZ 0.340 mm2), numerous microaneurysms (yellow circles) and venous tortuosity (yellow arrow)

**Fig. 7:**
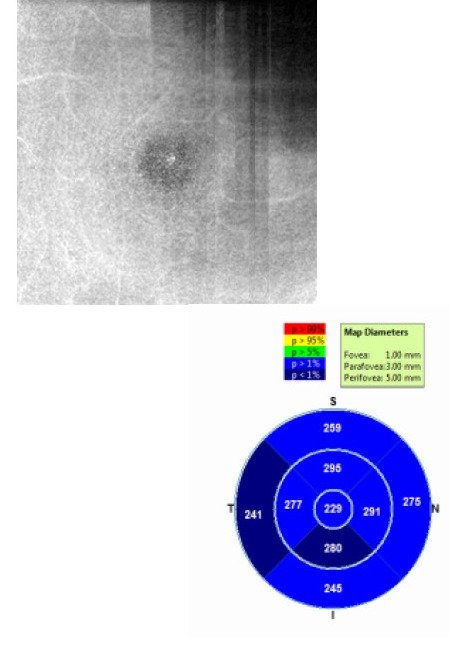
The OCTA en face image of the same patient where central subretinal fluid accumulation is observed but below the clinically significant threshold. Joined: the total macular volume SD-OCT measurement is normal

**Fig. 8:**
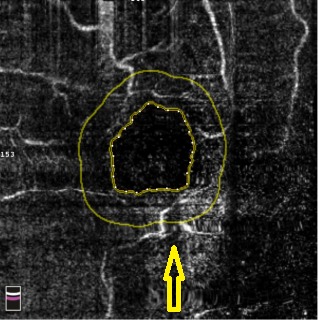
OCTA Avanti RTVue image of the foveal avascular zone of a patient with uncontrolled type 2 diabetes, proliferative diabetic retinopathy, and severe hypertension. There is a widening of the foveal avascular zone compared to normal value (FAZ 0.496 mm2) and lower parafoveolar a retinal neovascularization zone (yellow arrow). The normal architecture of the foveal area is altered with ischemic zones

## Discussion

FAZ measurements in the normal population conducted by Samara et al. found a population variability of 0.266 mm² ± 0.097 mm² and observed that its size was inversely correlated with the size of the macular cube and the macular thickness [**9**].

The decrease in tissue perfusion in diabetes cannot be detected in the incipient stages of diabetes. 

De Carlo et al. performed a 61-eyes OCTA study in patients with diabetes without clinically detectable diabetic retinopathy, in which they reported the presence of OCTA microvascular changes that correlated with FAZ widening to 0.348 mm2 versus control. Takase et al. reported statistically significant variations of FAZ compared to control in non-diabetic patients, and what drew further attention was that the increase in FAZ in diabetic patients was present regardless of the presence of clinical diabetic retinopathy as described in the ETDRS classification [**10**].

## Conclusions

Measurement of the foveal avascular zone area using OCTA early detects macular alterations that precede classical retinography and SDT OC determinations. 

Patients with mild and moderate diabetic non-proliferative retinopathy, associated with arterial hypertension, exhibited a minimal widening of the FAZ, but showed the tendency to be in a higher class of severity despite the relatively normal clinical aspect of the ETDRS classification. Patients with severe non-proliferative diabetic retinopathy or proliferative retinopathy associated with hypertension showed higher values of FAZ, and the qualitative analysis revealed large non-perfusion areas associated with neovascularization undetected by SDT.

**Disclosures**

None.

**Acknowledgment**

All authors have equal contribution to this paper.
